# Murine Leukemias with Retroviral Insertions at *Lmo2* Are Predictive of the Leukemias Induced in SCID-X1 Patients Following Retroviral Gene Therapy

**DOI:** 10.1371/journal.pgen.1000491

**Published:** 2009-05-22

**Authors:** Utpal P. Davé, Keiko Akagi, Rati Tripathi, Susan M. Cleveland, Mary A. Thompson, Ming Yi, Robert Stephens, James R. Downing, Nancy A. Jenkins, Neal G. Copeland

**Affiliations:** 1 Department of Medicine, Vanderbilt University Medical Center, Nashville, Tennessee, United States of America; 2 Department of Cancer Biology, Vanderbilt University Medical Center, Nashville, Tennessee, United States of America; 3 Mouse Cancer Genetics Program, National Cancer Institute, Frederick, Maryland, United States of America; 4 Department of Pathology, Vanderbilt University Medical Center, Nashville, Tennessee, United States of America; 5 Advanced Biomedical Computing Center, National Cancer Institute, Frederick, Maryland, United States of America; 6 St. Jude Children's Research Hospital, Memphis, Tennessee, United States of America; 7 Institute of Molecular and Cell Biology, Biopolis, Singapore, Singapore; University of Washington, United States of America

## Abstract

Five X-linked severe combined immunodeficiency patients (SCID-X1) successfully treated with autologous bone marrow stem cells infected ex vivo with an *IL2RG*-containing retrovirus subsequently developed T-cell leukemia and four contained insertional mutations at *LMO2*. Genetic evidence also suggests a role for *IL2RG* in tumor formation, although this remains controversial. Here, we show that the genes and signaling pathways deregulated in murine leukemias with retroviral insertions at *Lmo2* are similar to those deregulated in human leukemias with high *LMO2* expression and are highly predictive of the leukemias induced in SCID-X1 patients. We also provide additional evidence supporting the notion that *IL2RG* and *LMO2* cooperate in leukemia induction but are not sufficient and require additional cooperating mutations. The highly concordant nature of the genetic events giving rise to mouse and human leukemias with mutations at *Lmo2* are an encouraging sign to those wanting to use mice to model human cancer and may help in designing safer methods for retroviral gene therapy.

## Author Summary

Twenty patients with X-linked severe combined immunodeficiency (SCID-X1) have been successfully treated by gene therapy. Unfortunately, five of these patients have developed T-cell leukemia two or more years after receiving the therapeutic gene *IL2RG* on a retroviral vector. The leukemias developed because the vector inserted itself near cancer-causing genes and disrupted their normal regulation. Remarkably, in four patients, the vector inserted near a known T-cell oncogene, *LMO2*. We have found that in mice, similar retroviruses cause T-cell leukemias by inserting near *Lmo2*. We have found two leukemias that have retroviral insertions near *Lmo2* and *Il2rg* in the same cell. The probability of these insertions happening by chance is exceedingly small and these results imply that these two genes are deregulated together to induce leukemia. Our data show that *Lmo2* and *Il2rg* cooperate but may not be sufficient for leukemia development and additional mutations contribute to leukemia development. We have also found cooperating retroviral insertions in genes that are abnormally expressed in human T-cell leukemias. The mouse models provide unique insight into the pathogenesis of T-cell leukemia, and they are highly predictive of the leukemias caused by SCID-X1 gene therapy.

## Introduction

SCID-X1 patients are deficient in the common γ chain of the interleukin-2 receptor (*IL2RG*) [1]. In three SCID-X1 trials, CD34^+^ hematopoietic stem cells were cultured ex vivo and transduced with a defective Moloney murine leukemia virus (MuLV) expressing *IL2RG* and then transplanted back to the patients. The possibility that retroviral gene therapy could induce cancer through insertional mutagenesis had been widely discussed but until these trials no cases had been reported. Among the five leukemias that have occurred (four in the French and one in the UK trial), four had insertional mutations at *LIM domain Only 2* (*LMO2*) [2],[3],[4],[5]. *LMO2* is a T-cell oncogene [6], suggesting that these leukemias resulted from insertional mutagenesis. SCID-X1 is caused, in part, by a failure of T-cell production, and the infusion of immature gene-corrected CD34^+^ cells into SCID-X1 patients favors the engraftment of the T-cell lineage over other lineages. This could explain why T-cell leukemias predominate in these patients. *LMO2* is also expressed early in hematopoiesis [6],[7] which makes it a good target for insertional mutagenesis since retroviruses like to preferentially integrate near the 5′ end of actively transcribed genes [8]. *LMO2* is not the only T-cell oncogene expressed during early hematopoiesis, however, indicating there must be other reasons that favor mutagenesis of *LMO2*. A murine T-cell leukemia with insertional mutations at *Lmo2* and *Il2rg* has also been reported [9]. The probability of finding insertional mutations in both genes by chance in the same leukemia is exceedingly small and has led to the suggestion that *Il2rg* is a leukemia gene that cooperates with *Lmo2*. While *IL2RG* is not overexpressed in SCID-X1 leukemias [3] or in the mouse leukemia with an *Il2rg* insertion, subtle effects on its expression, such as an inability to downregulate its expression during T cell development, could be oncogenic [10].

Given the large number of *IL2RG*-infected cells transplanted into each patient, there is ample chance that a patient would receive a cell that contains an insertional mutation at *LMO2* [9]. Why then don't all patients develop leukemia? The most likely explanation is that other cooperating mutations are needed for leukemia to occur. Consistent with this, leukemias take several years to develop and contain mutations in other T-cell oncogenes [3],[11].

To provide further insights into this problem we cloned and sequenced the retroviral integrations from five murine leukemias containing insertional mutations at *Lmo2* using high-throughput ligation-mediated PCR (LM-PCR)/sequencing method that makes it possible to identify most of the insertionally mutated genes in these leukemias. We then compared the microarray data from human leukemias with upregulated *LMO2* expression and murine leukemias with insertional mutations at *Lmo2*. Our studies show that murine leukemias are highly predictive of the leukemias induced in SCID-X1 patients. They also support a model whereby deregulated *IL2RG* and *LMO2* expression cooperate to induce leukemia but are not sufficient and require other cooperating mutations.

## Results

### Five Murine Leukemias with Insertional Mutations at *Lmo2*

**Figure 1:**
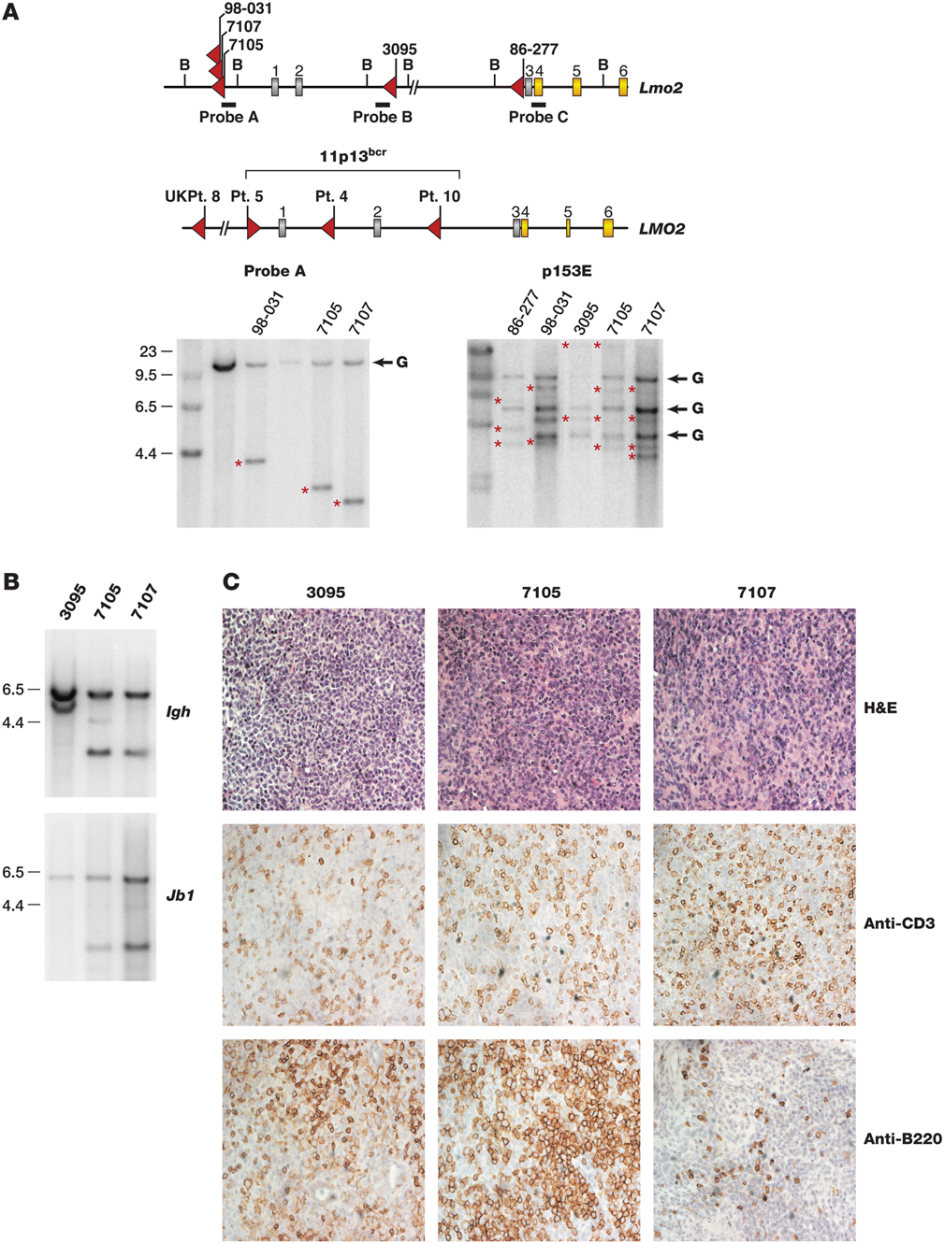
AKXD Leukemias have frequent clonal *Lmo2* insertions of T-cell origin. A) The mouse *Lmo2* gene is shown above the human gene and the five independent viral insertions cloned from AKXD tumors indicated with red arrows. Tumor names are shown above the arrows. The human *LMO2* gene has six exons; gray exons are noncoding and yellow are coding. The bracket indicates chromosomal breakpoints in T-ALL involving 11p13. *LMO2* was also insertionally activated in four SCID-X gene therapy patients. Only data for patients 4 and 5 from the French trial are available. Red arrows indicate the site and orientation of gene therapy vector insertion. The *Bam*HI sites used for Southern analysis and the positions of probes (A, B, C) are shown. Molecular weight markers are shown to the left of the blots. Retroviral insertions introduce *Bam*HI sites and show a rearranged band indicated with red asterisks. The germline bands were verified by Southern analysis of genomic DNA from brain and indicated G. The blot on the right was done using a probe (p15E) specific to the ecotropic envelope. B) Southern blots using probes specific for immunoglobulin heavy chain *Igh* and T-cell receptor *Jβ1* genes are shown with molecular weight markers. *Igh* shows a 6 kb germline band and the *Jβ1* blot shows a 5.8 kb germline band. The other bands indicate rearrangements that confirm the monoclonality of the tumors. C) Immunohistochemistry of *Lmo2*-clonal tumors 3095, 7105, and 7107 is shown. The H&E stain shows homogeneous appearing blast cells that have infiltrated and effaced the lymph nodes. All three tumors show mild CD3 staining and mild (3095) to moderate (7105) B220 staining.

The mouse Retroviral Tagged Cancer Gene Database (RTCGD) (http://rtcgd.abcc.ncifcrf.gov) lists four AKXD murine leukemias with insertional mutations at *Lmo2* [9], in addition to the AKXD leukemia analyzed previously which contains insertional mutations at *Lmo2* and *Il2rg*. All of the *Lmo2* insertions are located 5′ of coding exons 4–6 and are in the same general location as the insertions identified at *LMO2* in SCID-X1 patient leukemias and the chromosomal breakpoints at *LMO2* identified in sporadic human T-ALL [3],[12] (Figure 1A). Southern analysis showed these insertions are clonal (Figure 1A, bottom left panel, data not shown) and contain 2–4 clonal retroviral insertions each (Figure 1A, bottom right panel), as would be expected if they harbor mutations in *Lmo2*-cooperating genes.

Tumors 98-031 and 86-277 are T cell in origin [9]. Tumors 7105 and 7107 are also likely to be T cell as they have clonal *Igh* and T-cell receptor *Jβ1* rearrangements (Figure 1B). The origin of tumor 3095 is unclear. It was isolated from a mouse with thymomegaly and lymphadenopathy and contains *Igh* but no T-cell receptor gene rearrangements (Figure 1B, *Jβ2* not shown). Tumors 3095 and 7105 showed aberrant B220 staining (Figure 1C), which has been seen before in T-cell tumors from *Lmo2* transgenic mice [13] and in some human T-ALLs expressing B-cell markers [14].

### Large-Scale Cloning and Sequencing of Viral Integration Sites

To identify genes that might cooperate with *Lmo2* in tumor induction we adapted an LM-PCR method to amplify the other viral insertions present in the five AKXD leukemias [8],[15]. Following nested PCR using virus-specific and adaptor-specific primers, the amplified products were shotgun cloned. The inserts were then sequenced and BLAST-searched against the mouse genome and nearby candidate cancer genes identified (Table 1). These data were then combined with a less robust data set generated using inverse PCR (IPCR) [16]. This analysis identified 84 insertions in the five tumors (see Dataset S1). The percentage of cells in a tumor that harbor an insertion can be estimated from the number of shotgun clones isolated (Freq % in Dataset S1). Insertions present in all tumor cells will be enriched during PCR relative to insertions present in only a fraction of tumor cells and will thus be overrepresented in the shotgun library.

**Table 1:** Annotated common insertion sites found by LM-PCR/shotgun cloning of AKXD *Lmo2* tumors.

Tumor	Gene symbol	Protein function	Location	Distance	Dir
Tumor 3095					
	*Lmo2*	Transcriptional regulator	5 prime	36.35 kb	inv
	*Ccnd3*	Cell cycle regulator	5 prime	57.392 kb	inv
	*Mef2c*	Transcription factor	intron 2	not disrupt CDS	same
	*St3gal6*	Glycosyltransferase	intron 2	not disrupt CDS	inv
	*Tcf12*	Transcription factor	intron 5	disrupt CDS	same
Tumor 98-031					
	*Lmo2*	Transcription factor	5 prime	69.237 kb	inv
	*Il2rg*	Cytokine receptor	5 prime	6.244 kb	same
	*B3gntl1*	Glycosyltransferase	5 prime	0.739 kb	same
	*Mef2c*	Transcription factor	intron 2	not disrupt CDS	same
	*Bmi1*	Transcription factor	5 prime	67.614 kb	inv
	*Rap1gds1*	G-protein regulator	intron 2	disrupt CDS	inv
	*Sox4*	Transcription factor	exon 1	not disrupt CDS	same
Tumor 7105					
	*Lmo2*	Transcriptional regulator	5 prime	68.071 kb	inv
	*B3gntl1*	Glycosyltransferase	5 prime	13.488 kb	same
	*Me3*	Malic enzyme	intron 7	disrupt CDS	inv
	*Mef2c*	Transcription factor	intron 2	not disrupt CDS	same
	*Nmyc*	Transcription factor	3 prime	815 kb	same
	*Pou2f2*	Transcription factor	intron 1	disrupt CDS	inv
	*Prdm16*	Transcriptional regulator	intron 1	disrupt CDS	same
	*Sox4*	Transcription factor	exon 1	not disrupt CDS	same
	*Tcfe2a*	Transcription factor	5 prime	2.717 kb	inv
Tumor 7107					
	*Lmo2*	Transcriptional regulator	5 prime	68.387 kb	inv
	*Il2rg*	Cytokine receptor	5 prime	6.396 kb	inv
	*Il2rg*	Cytokine receptor	5 prime	6.759 kb	inv
	*Irs2*	Adaptor for signal transduction	intron 1	disrupt CDS	same
	*Irs2*	Adaptor for signal transduction	intron 1	disrupt CDS	same
	*Mef2c*	Transcription factor	5 prime	389.868	inv
	*Ccnd1*	Cell cycle regulator	5 prime	110.927 kb	inv
	*Fgfr3*	Growth factor receptor	3 prime	8.94 kb	inv
	*Laptm5*	Lysosomal protein	intron 1	disrupt CDS	inv
	*Prdm16*	Transcriptional regulator	intron 1	disrupt CDS	same
	*Rere*	Transcriptional regulator	N/D	N/D	
Tumor 86-277					
	*Lmo2*	Transcription factor	5 prime	0.955 kb	inv
	*Fli1*	Transcription factor	intron 1	disrupt CDS	same
	*Fnbp4*	Cytoskeletal protein	5 prime	0.443 kb	inv
	*Sox4*	Transcription factor	5 prime	38.481 kb	inv

Genes nearest the sites are shown below the tumor names. Predicted or known protein function and the distance and orientation of the insertions with respect to the nearby gene are shown. All these insertions are CIS in the RTCGD (see text).

Remarkably, 35 insertions are located at common insertion sites (CISs), a highly significant result (Fisher's exact test p = 7.4×10^−39^) (Table 1). CISs are regions in the genome that are mutated by viral insertion at a rate higher than predicted by random chance and are thus likely to harbor a cancer gene [16]. Thirty-one of these genes are also mutated in human cancer, another highly significant result (Fisher's exact test p = 5.7×10^−37^) (Table S1, Dataset S1) [17]. This strongly suggests that some of these genes are *Lmo2*-cooperating genes.

### A Second Tumor with an Insertion at *Il2rg*

**Figure 2:**
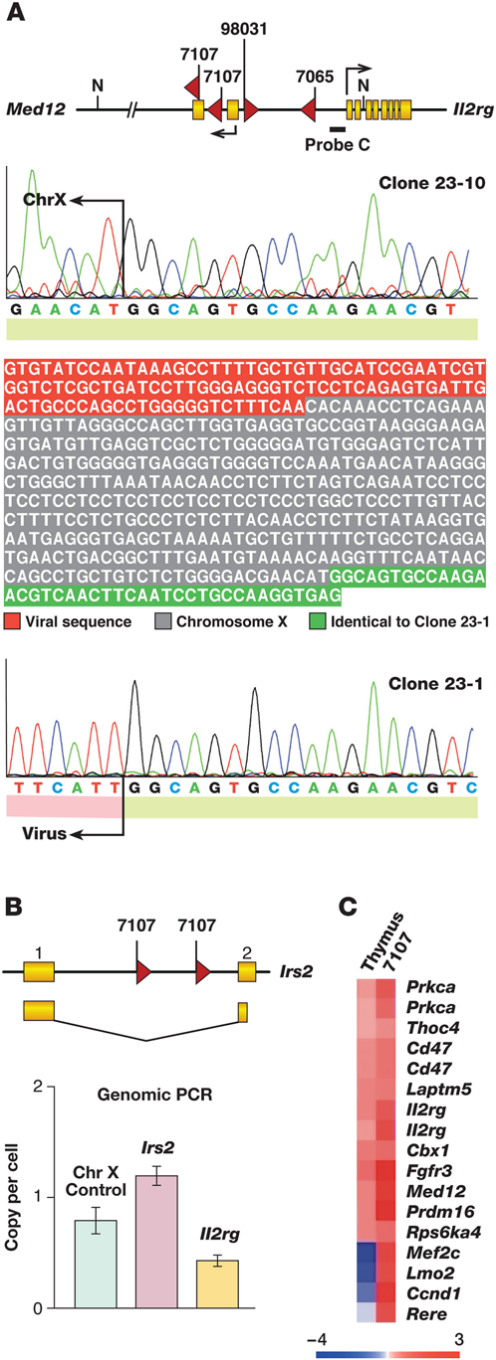
Tumor 7107 has two independent insertions in *Il2rg* and *Irs2* genes. A) LM-PCR identified two independent insertions in tumor 7107, 5′ of the eight coding exons of the *Il2rg* gene. The two viral chromosomal junctions were independently cloned and sequenced. The insertions were 396 bp apart and should be present in the same clone if they occurred on the same chromosome. Partial sequence for clone 23-10 shows the region of identity with clone 23-1 (green). For clone 23-1, we found viral sequence 5′ of this region instead of chromosome X genomic sequence. B) Two insertions in the *Irs2* gene were also isolated from the same tumor. Primers were designed for one allelic insertion for *Irs2* and *Il2rg* and real time PCR performed on the genomic DNA. Primers for a chromosome X sequence were used as reference and show a copy number of 1 for this male mouse. As shown, each insertion comprises about 50% of total tumor genomic DNA. We ruled out polyploidy for *Irs2* and *Il2rg* genes (see Figure S4). C) Panel shows a heat map of gene expression data using the mouse Affymetrix array. The expression of genes near high frequency insertions cloned from 7107 is shown with respect to normal thymus. Log_2_ intensity scale is shown under the heat map.

Surprisingly, tumor 7107 contains two *Il2rg* insertions (Table 1), which was confirmed by conventional cloning and sequencing (Figure 2A). Thus, 2 of the AKXD murine leukemias with insertions at *Lmo2* also contain insertions at *Il2rg*, a highly significant result (p = 1.34×10^−9^, see Text S1 for calculation). Likewise, tumor 7107 has two insertions at *Irs2* (Figure 2B), another highly significant result (p = 1.16×10^−5^, see Text S1 for calculation). Il2rg and Irs2 are functionally linked in lymphoid cells where it has been shown that Il2rg can promote the phosphorylation of Irs2 by binding to and activating the tyrosine kinase Jak3 [18], which may explain the co-selection for mutations in these genes. To quantitate the *Irs2* and *Il2rg* insertions, we amplified one *Lmo2* and one *Il2rg* insertion using insertion-site-specific primers and real time PCR. The *Irs2* insertion was present at about one copy per cell (Figure 2B), indicating it is present in every tumor cell. The other *Irs2* insertion must therefore be in the same tumor cell, either on the same or different chromosome. In contrast, the *Il2rg* insertion was present at 0.5 copies per cell (Figure 2B), suggesting it is present in only half the tumor cells of this male mouse. We ruled out hyperploidy for the *Irs2* locus and also confirmed that the *Il2rg* gene was present at one copy per cell (see Figure S4). The two *Irs2* insertions must therefore have occurred first and in the same tumor cell followed by the two *Il2rg* insertions in different subpopulations of tumor cells. Similar to what was reported previously [9], *Il2rg* is not misexpressed in tumor 7107 (see Figure S3). Most of the genes that were insertionally mutated in this tumor were also highly overexpressed when compared to normal thymus control (Figure 2C). Since our prior study, exons for the *Med12* gene were annotated and are shown to direct transcription in the opposite orientation to *Il2rg* (see Figure 2A). All the tumors have insertions in the same orientation to *Med12* except tumor 98-031 and one of the insertions disrupts the second coding exon of *Med12*. *Med12* was not found to be up-regulated in the tumors and no spliced fusion transcript between provirus and *Med12* could be identified (see Figure S3). It is conceivable that these insertions implicate *Med12* in tumorigenesis and not *Il2rg*, however, the RTCGD contains many common insertion sites in genes of the *Il2rg* pathway (e.g. *Il2ra*, *Il4ra*, *Il7*, *Jak1*, *Stat5a/5b*).

### Other Multiply Mutated Genes

**Figure 3:**
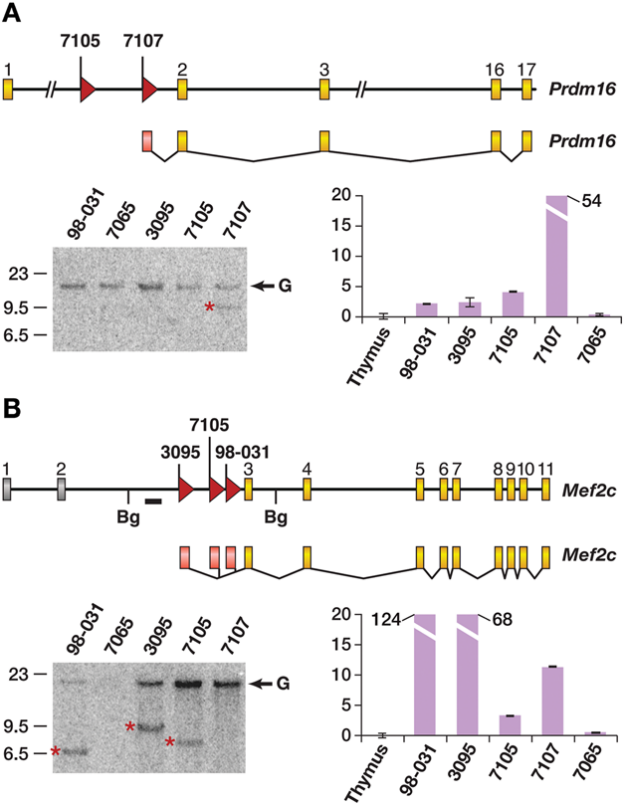
*Prdm16* and *Mef2c* genes are frequently insertionally mutated in AKXD *Lmo2* tumors. A) The *Prdm16* gene was targeted in tumors 7105 and 7107. Coding exons are in yellow. Tumor 7107 had a clonal insertion as shown by the Southern blot. G shows germline bands and a red asterisk shows the rearrangement. The graph shows quantitative RT-PCR analysis for *Prdm16*. Real time PCR was normalized to expression in normal thymus. Tumor 7107 had a viral fusion transcript spliced from the 5′LTR to exon 2. The transcript from tumor 7105 was not recovered from the RNA since the insertion was subclonal. B) The *Mef2c* gene was insertionally activated in three tumors, 3095, 7105, and 98031. Southern blot using *Bg*lII-digested genomic DNA using the probe (thick line) shows all insertions were clonal and early in tumorigenesis. Quantitative RT-PCR shows the up-regulation of *Mef2c*.

Several other genes are also mutated more than once in the *Lmo2* tumors, indicating that they might also represent *Lmo2*-cooperating genes. Two tumors have insertions in the leukemia transcription factor oncogene *Prdm16* (Figure 3A) but only the insertion in tumor 7107 is clonal (Figure 3A, bottom left panel). Quantitative RT-PCR/sequencing revealed marked upregulation of a *Prdm16* fusion transcript in tumor 7107 that initiates in the viral 5′LTR and splices to *Prdm16* exon 2 (Figure 3A, bottom right panel). This transcript is predicted to encode a truncated protein similar to that expressed in human leukemias with *PRDM16* mutations [19].

Remarkably, 3 tumors also have insertions in intron 2 of *Mef2c* (Figure 3B, top panel), which is a transcription factor oncogene that cooperates with *Sox4* in leukemia induction [20]. All insertions are in the same transcriptional direction as *Sox4* and are clonal (Figure 3B, bottom left panel). Quantitative RT-PCR/sequencing revealed high expression of *Mef2c* fusion transcripts in these tumors. The transcripts all initiated in the viral 5′LTR and spliced into the first coding exon of *Mef2c* (Figure 3B, bottom right panel), similar to what is reported for other leukemias with *Mef2c* insertions [20]. Tumor 7107 also showed high *Mef2c* expression (Figure 3B, bottom right panel) and has an insertion located 390 kb upstream of *Mef2c* (Table 1). There is precedent for long-range enhancer effects exerted by proviruses [21],[22], potentially accounting for the high *Mef2c* expression. Thus, 4 of 5 AKXD tumors have insertions at or near *Mef2c*, strongly suggesting that *Mef2c* is an *Lmo2*-cooperating oncogene. Consistent with this, tumors 98-031, 7105 and 86-277 also had insertions near the *Mef2c*-cooperating gene *Sox4*, although only the insertion in tumor 7105 was clonal (data not shown).

Finally, tumors 98-031 and 7105 have insertions in the 5′ end of the putative acetylglucosaminyltransferase gene, *B3gntl1* (Table 1). Very little is known about this gene so the significance of this result is unclear.

### Mutations in E2A-Related Genes

**Figure 4:**
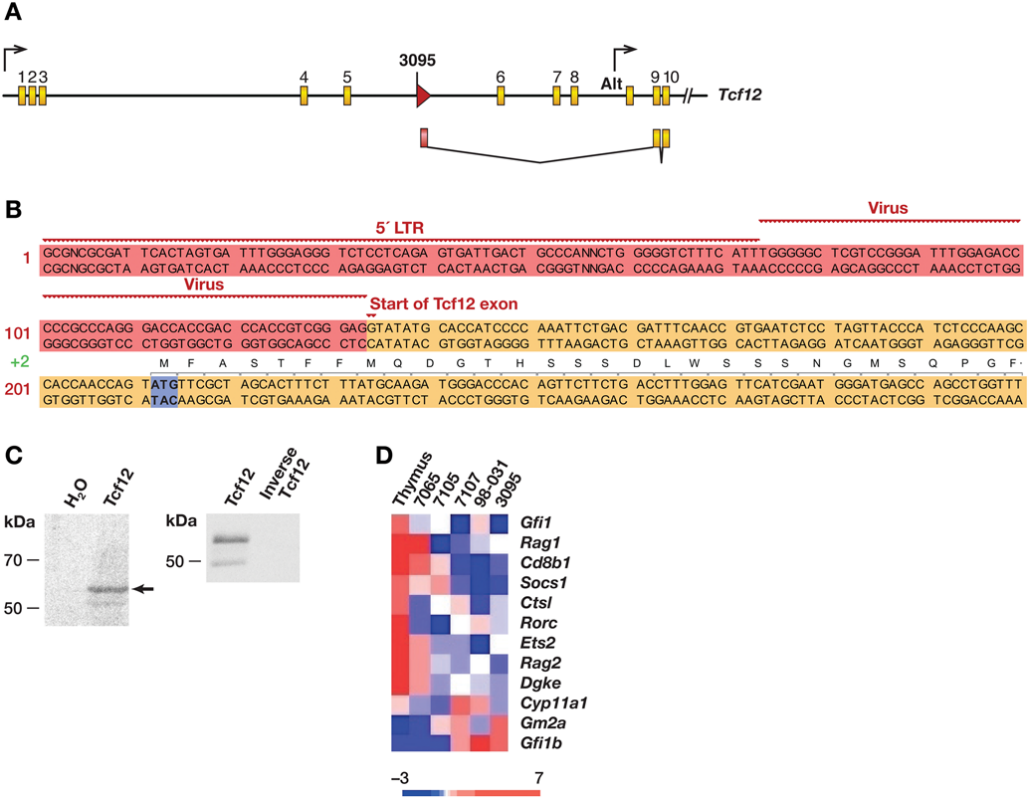
*Tcf12* insertion creates a truncated protein. A) Top panel shows the mouse *Tcf12* gene with arrows indicating the ubiquitous promoter and an alternate promoter found in thymus (Alt). Yellow exons are coding and the red arrow indicates the retroviral insertion cloned from tumor 3095. B) Panel shows the sequence of the viral fusion transcript cloned by RT-PCR from this tumor. 5′RACE showed transcription was initiated in the viral 5′LTR and spliced into exon 9. The partial sequence of the viral fusion transcript is shown. Red indicates viral sequences, LTR and 5′ of gag. Yellow denotes the start of exon 9 of *Tcf12*. The first ATG codon (blue) is in frame with the rest of *Tcf12*. C) Panel shows two SDS-PAGE blots: left panel shows in vitro transcription and translation of the cloned viral fusion cDNA with radiolabeled ^35^S-methionine. A strong band at 55 kDa appears as well a weaker band at 50 kDa. The in vitro translated proteins were derived from Tcf12 since they were immunoprecipitated with an antibody specific to the protein's COOH-terminus (right panel). D) This heat map shows microarray analysis of the *Lmo2*-clonal tumors with selected E2A targets. The genes shown are normally up-regulated by E2A except for *Gm2a* which is repressed. Included in the comparison is tumor 7065 which had a clonal insertion in *Notch1* and no up-regulation of *Lmo2*.

We also identified insertions in two E2A-related genes, *Tcfe2a* (*TCF3* in humans) and *Tcf12* (Table 1), suggesting a role for E2A-related genes in the *Lmo2* tumors (p = 0.008). *TCF3* is translocated to a number of fusion partners in pre- and pro-B-ALL [23], while *TCF12* is fused to *NR4A3* in extraskeletal mixoid chondrosarcoma [24]. The *Tcf12* insertion is located in intron 5 and is in the same transcriptional orientation as *Tcf12* (Figure 4A). 5′RACE showed that it induces a fusion transcript, which initiates in the 5′LTR and splices to *Tcf12* exon 9 (Figure 4B). The first ATG is located in exon 9 and is in-frame with *Tcf12* coding sequences. Assuming this is the preferred translational start site, the predicted polypeptide would lack 219 residues from the amino terminus, which was confirmed by *in vitro* transcription and translation studies (Figure 4C, left panel). The major polypeptide migrated at 55 kDa, similar to its predicted mass of 52 kDa. A faint 50-kDa band was also observed. Both proteins were immunoprecipitated with a rabbit polyclonal antibody specific to Tcf12, confirming they are Tcf12-derived (Figure 4C, right panel). The smaller protein could arise from an alternate translational initiation site or be due to protein degradation.

Tcf12 and Tcfe2a are class I bHLH transcription factors. The amino terminus of Tcf12 encodes two transactivation domains, AD1 and AD2. AD1 is similar to AD1 of the related E2A proteins, which has been shown to have greater potency in transactivation than AD2 [25]. While there are no mutagenesis data on the homologous domains of Tcf12, the truncated *Tcf12* transcript expressed in tumor 3095 would lack the AD1 domain but have an intact AD2 domain, which could result in attenuated transactivation.

Microarray analysis showed there was considerable differential expression of E2A target transcripts when the five tumors were compared to normal thymus [26] (Figure 4D). Similar, but somewhat less, differential expression, was observed when the tumors were compared with tumor 7065, which has an insertion at *Notch1* (Figure 4D). With the exception of *Gfi1b*, targets activated by *E2A* are poorly expressed as expected if E2A signaling is attenuated. Conversely, *Gm2a*, which is repressed by *E2A*, was highly expressed. These results provide further evidence that the E2A pathway is attenuated in tumors with mutations at *Lmo2*.

Microarray expression analysis also showed that these tumors express *Tal1* as well as the other experimentally verified Lmo2 binding partners, consistent with the involvement of *Lmo2* in these tumors (Figure S1).

### Insertionally Mutated Genes Are Highly Expressed in Human T-ALL with High *LMO2* Expression

To provide additional evidence that the genes insertionally mutated in murine *Lmo2* tumors are causally associated with human T-ALL, we examined the raw data from three large human T-ALL microarray data sets (total of 118 cases) [27],[28],[29] and asked whether any of the murine genes are deregulated in human T-ALL with high *LMO2* expression. We separated the human T-ALL cases into *LMO2*-high and *LMO2*-low expression classes and performed clustering analysis to identify the genes differentially expressed in the *LMO2*-high class (p<0.001 significance level). The data sets were remarkably consistent in the genes that were upregulated in the *LMO2*-high class, despite different array platforms and patient heterogeneity. When genes that were consistently deregulated in the *LMO2*-high class were queried against the RTCGD, a statistically significant number were located at CISs or are insertionally mutated in murine tumors with *Lmo2* insertions (Figure S2), and the number of such genes is much higher than expected by chance (Figure S2; Ferrando et al, p = 1.1×10^−3^ and Yeoh et al, p = 1.1×10^−3^; Chiaretti et al, p = 4.1×10^−6^).

**Figure 5:**
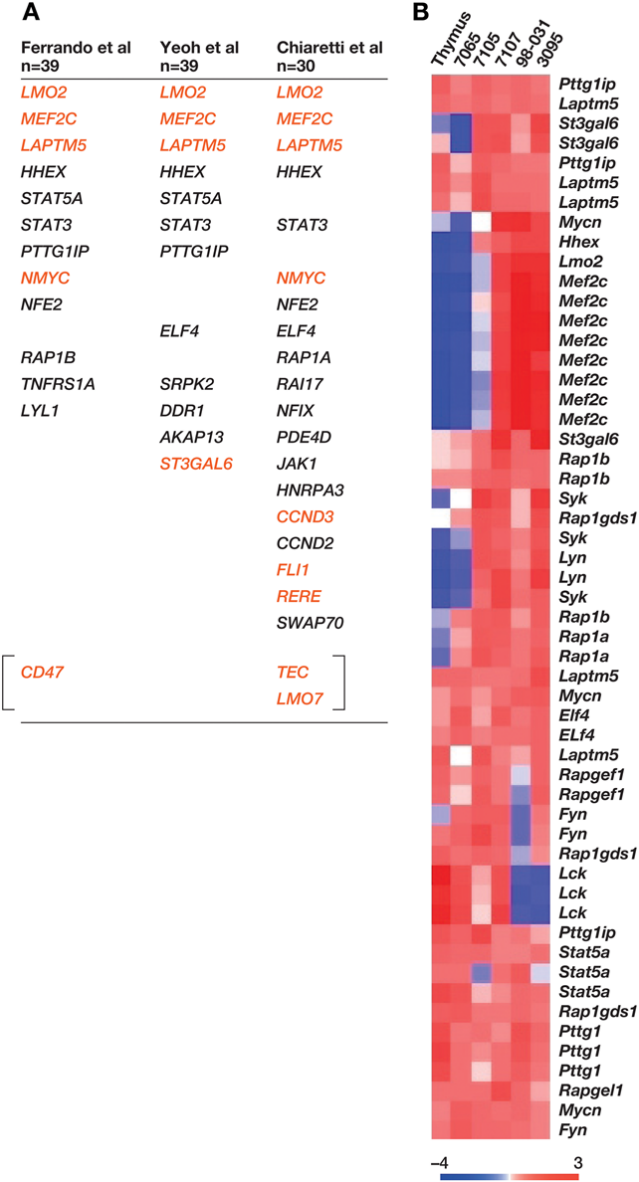
Human microarray studies show consistent high-level expression of genes and pathways insertionally mutated in AKXD *Lmo2* tumors. A) Genes selectively expressed in *LMO2*-overexpressing patients from three human microarray analyses are shown. Below the first author citations are shown the number of patients in each study. The genes shown in red are retroviral insertions in the mouse *Lmo2*-clonal tumors. B) Select genes that were expressed in the *LMO2*-high class of patients were probed in the mouse tumors and are shown in the heat map. Several of these genes (e.g. *Mef2c*, *Hhex*, *Syk*, *Lyn*, *Mycn*) are differentially expressed in the mouse tumors in comparison to normal thymus and tumor 7065.

Remarkably, *MEF2C* was overexpressed in the *LMO2*-high group in all three T-ALL data sets (Figure 5A). This is extremely unlikely to have occurred by chance and further confirms the role of this gene in human T-ALL. Likewise, *LAPTM5*, which is the site of an insertion in tumor 7107, was identified in all three data sets, while *NMYC*, an insertion site in tumor 7105, was identified in two microarray data sets (Figure 5A). *STAT5A* was found in two data sets. *Stat5a* is not insertionally mutated in the murine tumors but it is a validated leukemia gene and functions in the same signaling pathway as *IL2RG*. Most of the genes that clustered with the *LMO2*-high class were also highly expressed in the mouse tumors and some showed differential expression in comparison to normal thymus or tumor 7065 (Figure 5B).

Multiple *RAP1* pathway genes also clustered with the *LMO2*-high group, including *RAP1B*, *RAP1A* and *RAPGEF5*. None are insertionally mutated in AKXD tumors, however, *Rap1gds1* is insertionally mutated in tumor 98-031 and is the site of a recurrent chromosomal translocation in human T-ALL [30]. Rap1gds1 stimulates the exchange of GTP for GDP on Rap1 and is an activator of the Rap1 pathway. Upregulation of *Rap1gds1* could therefore produce the same effect as upregulating *RAP1B*, *RAP1A* or *RAPGEF5*. Pathway analysis on genes shared in at least two microarray datasets (mouse and human combined) also suggested a role for cytokine signaling in tumor development (see Figure S5).

## Discussion

To provide a better mechanistic understanding of SCID-X1 patient leukemias, we have analyzed five murine T-ALLs with insertional mutations at *Lmo2*. In each case these insertions are clonal. Therefore, they must have occurred early in tumor induction, similar to four of the five cases of gene therapy-induced leukemias. Transcriptional profiles of these T-ALLs showed high expression of the *Tal1* and *Lyl1* oncogenes as well as other Lmo2-binding partners such as Gata1 and Gata2. *TAL1* and *LYL1* are class II bHLH transcription factors that are frequently overexpressed along with *LMO2* in human T-ALLs [27],[31]. Consistent with this, patient #4, who developed leukemia in the French trial, and patient #8, who developed leukemia in the English trial, showed rearrangement of the *SIL-TAL1* loci along with insertional activation of *LMO2* [3],[5],[32].

Murine T-ALLs with *Lmo2* insertions also showed consistent downregulation of genes activated by E2A and upregulation of genes repressed by E2A, consistent with previous studies suggesting that Lmo2 redirects E2A activity by binding it through its partner proteins, Tal1 or Lyl1 [6]. Genes that are activated by the Lmo2/Tal1/E2A/Gata1-containing complex in normal erythrocyte development were also overexpressed in one murine T-ALL with high Gata1 expression. These findings suggest that murine T-ALLs with *Lmo2* insertions resemble human T-ALLs that are initiated by *LMO2* deregulation.

Our high throughput insertion site analysis identified additional disease-related genes that are likely to cooperate with *Lmo2* in leukemia induction. We identified a second tumor (7107), in addition to tumor (98-031) described previously [9] that has an *Il2rg* insertion. Remarkably, this tumor has two insertions at *Il2rg* that occurred independently suggesting strong selection for *Il2rg* deregulation in *Lmo2*-initiated T-ALL. In addition, tumor (7065) has insertions in *Notch1* and *Il2rg*, suggesting that *Il2rg* might be able to cooperate with other T-cell oncogenes (i.e. *Notch1*) in leukemia induction.

The newly identified insertions are also close to or within the *Med12* gene. Similar to *Il2rg*, this gene is not activated or downregulated by the insertions. We favor *Il2rg* as the retroviral target since the RTCGD is replete with common insertions in cytokine receptors that act in the exact same pathway as *Il2rg*. More recently, we have found *Il2rb* is a common insertion site in a retroviral insertional mutagenesis screen in *Lmo2* transgenic mice (U.P. Davé, unpublished observation). Other Il2rg binding partners, Il7ra and Jak1, were insertionally activated in the same screen which has not reached saturation. While still somewhat controversial, two studies provide additional evidence that *Il2rg* can function as an oncogene under some circumstances [33],[34]. A statistically significant enrichment of Il2rg-dependent cytokine pathways in murine T-ALLs containing *Lmo2* insertions and human *LMO2*-overexpressing T-ALLs was also observed by Ingenuity pathway analysis (see Figure S5). A role for these pathways in T-ALL is further supported by studies showing that cytokines, which depend on Il2rg for signaling can induce T-ALL in transgenic mice, and by the murine T-ALL with two *Il2rg* and two *Irs2* insertions. This murine T-ALL is significant since *Irs2* encodes a protein adaptor that is phosphorylated by JAK3 tyrosine kinase in response to cytokine ligation by Il2rg and its heterodimeric partners such as Il7ra, Il4ra and Il9ra [18],[35]. The occurrence of leukemias in the SCID-X1 gene therapy trials also lends compelling support to the notion that *IL2RG* is oncogenic and a cooperating “hit” with *LMO2*.

*Il2rg* expression was not upregulated in any of these three tumors but there is precedent for cancer genes being dysregulated without gross overexpression or at specific developmental stages [36],[37],[38]. Similarly, the *IL2RG* transgene was not overexpressed in patients receiving gene therapy or in those developing T-ALL [5],[11]. We theorize that a gene is less able to be silenced if there is a nearby retroviral insertion. Enforced expression of *Il2rg* has been shown to cause T-cell leukemias without gross overexpression [33],[39]. In the study by Woods et al, they specifically remark that transgene levels in thymic lymphomas were comparable to *Il2rg* expression levels seen in developing thymi [34]. Another compelling possibility is that *Il2rg* is misexpressed in a target cell population where *Il2rg* is not normally found such as a cell more primitive than a thymocyte precursor. The study by Shou et al presents an alternate hypothesis that the SCID-X1 background is required for leukemia development perhaps due to altered numbers of hematopoietic precursors or stem cells [33]. It may be that the lack of *Il2rg* creates a differentiation block that expands a cell type that is susceptible to transformation. Recurrent insertions in genes that are also CIS in the RTCGD were identified by Shou et al but *Lmo2* was not one of them. The tumors were not analyzed for *Lmo2* overexpression or for *Notch1* activating mutations. Our data suggests that leukemia requires many “hits” and in their model somatic mutation in cooperating oncogenes or tumor suppressor genes may be more likely than insertional mutation since their vector was replication-defective.

Retroviral insertion site profiles have recently been published for the French and English cohorts of SCID-X gene therapy patients (n = 14) [4],[5],[11],[40]. Presently, there is no statistical difference between the incidence of leukemia in the French (4 of 10) and English patients (1 of 10) and with long term follow-up, even the insertion site profiles may be quite similar in the two studies. Comparison of the insertion sites identified in the French patients with those in RTCGD showed there was a statistically significant number of insertions in these patients, which are located at CISs in murine hematopoietic tumors (63/554, Fisher's exact test, p = 4.5×10^−22^). Remarkably, patient #10 of the French trial showed clonal insertions in *LMO2* and *BMI1*; these two insertions were observed in tumor 98-031, underscoring the predictive power of our mouse models [11].

In designing safer vectors for transduction, it will be important to consider self-inactivating LTRs and perhaps lentiviral backbones, which have different insertion site preferences than gamma-retroviruses, which may carry a lower risk of insertional mutation [41]. With the high transduction efficiencies achieved in these gene therapy trials, one could also see clonal expansion in cases where the transduced gene is not oncogenic. This, in fact, happened recently in two chronic granulomatous disease patients treated by retroviral gene therapy [42]. In both patients, a clonal expansion of the myeloid compartment was observed that began 3 to 5 months post-transplant. Clonal expansion was associated with insertional mutation of just three genes: *MDS/EVI1*, *PRDM16*, or *SETBP1*. Astonishingly, in the mouse, insertional mutation of these same genes has been associated with immortalization of early hematopoietic progenitor and/or myeloid progenitor cells [19]. Insertional mutations in these genes may have been selected in transplant patients due to their effects on increased self-renewal or engraftment potential. *Prdm16* is also insertionally mutated in two of the murine T-ALLs with *Lmo2* insertions so this gene's involvement in tumor induction might not be limited to the myeloid lineage.

## Materials and Methods

### Ethics Statement

Mice were aged in SPF facilities according to approved IACUC protocols at NCI-Frederick.

### Mice, Tumors, and DNA/RNA Preparation

Various AKXD strains were developed at the Jackson Laboratory. These are recombinant inbreds that have a high spontaneous rate of leukemia or lymphoma onset due to the presence of an endogenous retrovirus [43],[44]. The mice are viremic from birth and perhaps in utero and develop disease at various latencies, usually over six months of age. Murine leukemia viruses are not introduced. The mice are simply aged in SPF facility until the onset of disease. At the first appearance of morbidity, mice were sacrificed and gross necropsies performed. Organomegaly and abnormal features were noted and lymphoid and hematopoietic tissue was harvested and flash frozen in liquid nitrogen. Tissue was also fixed in formalin for immunohistochemistry. Frozen tissue was used in the preparation of high molecular weight DNA and whole RNA as previously described [45].

### Tumor Histochemistry and Southern Analysis

Tumor genomic DNA was restriction digested and loaded on to agarose gels for overnight runs. The gels were transferred as described to Nitrocellulose (Amersham) [44]. Membranes were baked and UV-crosslinked. They were hybridized with ^32^P-labeled probes and exposed to film. For quantification, blots were exposed to phosphorimager plates and a Fuji phosphorimager used for quantitation. Details on probes used are available upon request. Paraffin-embedded tumors were sectioned and probed with a labeled BAC encompassing the mouse *Irs2* gene. Labeling and FISH were performed by manufacturer's protocol (Vysis).

### Retroviral Insertion Site Cloning and Analysis

Inverse PCR was performed as previously described [46]. For LM-PCR, tumor genomic DNA (1 µg) was digested overnight with *Nla*III or *Mse*I. The internal amplicon was eliminated by a *Spe*I digest. DNA was column purified (Amersham GFX) and ligated to a double stranded adaptor. This adaptor was made by annealing sense and antisense strands with *Nla*III or *Mse*I compatible overhangs as previously described [45]. The ligation mix was column purified (Qiagen) and then used for nested PCR. The PCR products were column purified and then ligated into pGEMT-Easy (Promega) plasmids and plated. Ninety six colonies were picked and miniprep DNA prepared using Whatman Ultra filters. Miniprep DNA was sequenced in 96 well format (Functional Biosciences) and files were analyzed in FASTA format. Valid sequences contained 3′LTR and adaptor at either end were then batch BLASTed against the mouse genome. Annotated insertions are posted at http://rtcgd.abcc.ncifcrf.gov. Insertions were confirmed by either Southern analysis or PCR using gene-specific primers in the vicinity of insertions. For tumor 7107, gene specific primers corresponding to *Il2rg* and *Irs2* were used in real time PCR (Biorad MyIQ) to confirm biclonality. For tumor 3095, virus-specific and *Tcf12*-specific primers were used in RT-PCR to generate cDNA for the viral-fusion transcript. 5′RACE was done using SMART Race kit (BD Biosciences) to verify that the transcriptional start site was in the virus. The full length cDNA was sequenced from both ends and TA-cloned into pGEMT-Easy (Promega); *Not*I digest released this cDNA for subcloning into pcDNA3.1 (Invitrogen). Orientation was verified and *in vitro* transcription and translation was performed using the TnT kit (Promega) with ^35^S-labeled methionine (Amersham). In vitro translated Tcf123095RIS was resuspended in RIPA buffer and immunoprecipitated using antibody against the COOH-terminus of Tcf12 (sc-357, Santa Cruz Biotechnology).

### Gene Expression Analyses of Mouse Tumors

Tumors were homogenized in Trizol reagent (Sigma) and whole RNA was isolated by manufacturer's protocol. First strand cDNA was synthesized using oligo-dT primer and Superscript II or III reverse transcriptase enzymes (Invitrogen). Primers used for RT-PCR are available upon request. For real time PCR, we used Biorad's MyIQ or ABI 7700 machines with either Sybr green master mixes (Biorad) or Taqman probes (Applied Biosystems). Probe assays used are available upon request and were validated by Applied Biosystems. RNA was processed for hybridization to the Affymetrix chips according to standard protocols (see Text S1).

### Microarray Analyses of Human T-ALL Datasets

Microarray analysis was performed with BRB-ArrayTools (version 3.2.3) developed by Biometric Research Branch at the National Cancer Institute. *LMO2*- positive and *LMO2*-negative tumors were compared by using univariate significance tests at the significance level of 0.001. The maximum false discovery proportions were restricted to 0.1 using multivariate permutation tests. As for the criteria of *LMO2*-positive and *LMO2*-negative tumors, there was no clear cut-off level for classifying a sample as having either high or low expression. We used artificial cut-off values to classify tumors into two groups. At first, we identified the median value of *LMO2* expression signals as an initial indicator for the classification, and then we removed the marginal cases from the comparisons. For example, in the case of St Jude's data, we classified tumors with *LMO2* signal>2000 as *LMO2*-positive group (13 cases), tumors with *LMO2* signal<1000 as *LMO2*-negative group (14 cases).

Statistical comparison between tumor classes was done using the “BRB Array Tools” software (http://linus.nci.nih.gov/BRB-ArrayTools.html). We collated CEL file format of Affymetrix data by using the ‘RMA’ method [47] of the ‘affy’ package from BioConductor (http://www.bioconductor.org/). To identify genes that were differentially expressed among the two classes, we used a random-variance t-test. The random-variance t-test is an improvement over the standard separate t-test as it permits sharing information among genes about within-class variation without assuming that all genes have the same variance [48]. Genes were considered statistically significant if their p values were less than 0.001. A stringent significance threshold was used to limit the number of false positive findings. We also performed a global test of whether the expression profiles differed between the classes by permuting the labels of which arrays corresponded to which classes. For each permutation, the p values were re-computed and the number of genes significant at the 0.001 level was noted. The proportion of the permutations that gave at least as many significant genes as with the actual data was the significance level of the global test. We performed cluster analysis of genes and produced a heat map image to represent the over- and under-expression of each gene in each sample. We used quantile data ranges to ensure the even presence of all available colors on the map.

For comparison of mouse and human datasets, we used Fisher's exact test, binomial distribution and Chi-square tests to generate p values. Please see Text S1 regarding specific statistical questions analyzed.

## Supporting Information

Figure S1 AKXD *Lmo2* tumors co-express *Tal1*. A) quantitative RT-PCR shows *Lmo2* and *Tal1* are overexpressed in the tumors with respect to normal thymus. To the right are two heat maps from microarray expression data. B) This panel shows T-cell leukemia transcription factors and their expression in the *Lmo2*-clonal tumors. RNA from normal thymus and tumor 7065, which has a clonal, activating mutation in *Notch1*, are included in the comparison. *Lmo2*-clonal tumors have higher class II bHLH transcription factor expression and lower expression of *E2A* genes when compared to normal thymus and tumor 7065. C) This heat map shows documented *Notch1* targets and their expression in the same tumors. All genes shown are normally up-regulated by *Notch1* except for *Ebf1*, *Sfpi1*, and *Flt3*, which are repressed. The *Lmo2*-clonal tumors show low expression of *Notch1* target genes and higher expression of genes that are repressed. Tumor 98031 has higher expression of *Hes1* and *Il2ra* and lower expression of *Ebf1*, consistent with a heterozygous mutation in the heterodimerization domain of *Notch1*. The rest of the tumors had wild type *Notch1* sequences. D) Experimentally confirmed Lmo2-binding partners are shown on this heat map. All of these were expressed at levels higher than normal thymus and tumor 7065. The *Lmo2*-clonal tumors had high expression of *Gata1* and *Gata2*. *Gata1* was very high in tumor 98031 and so we assayed the expression of erythroid genes in this tumor. E) Numerous erythroid genes were up-regulated in 98031. The genes denoted by the black arrows are activated by an Lmo2/Gata1/2/Tal1/E47/Ldb1-containing oligomeric complex that binds to promoter E box/Gata-motifs. Log2 intensity scales are shown for the heat maps.
(2.29 MB TIF)

Figure S2 Human T-ALL microarray analysis shows representation of many CIS in the transcriptional profile of *LMO2*-overexpressing patients. Raw data were retrieved from three large published T-ALL studies and cases were clustered into *LMO2*-high expressing and *LMO2*-low expressing classes. We next sought to find the most statistically significant genes that clustered with the *LMO2*-high group. The studies' first authors are cited as well as the platforms used. In parentheses, we show how many probes were on the chip used. Below this, we show the number of CIS divided by the total number of genes that clustered with the *LMO2*-high expressing cases. The last number is the p value for identifying these CIS as calculated by Fisher's exact test.
(2.78 MB TIF)

Figure S3 *Il2rg* insertions are clonal but cause no transcriptional activation. A) the structure of the genomic region with *Il2rg* and *Med12* genes is shown. Yellow exons are coding and black arrows show transcriptional start sites and orientation. Southern analysis of tumor 7107 was done using *Nco*I digested tumor DNA. The germline band is identified by the arrow, G. The rearranged band is present with equal intensity and shown by the red asterisk. The size of the *Nco*I fragment did not allow discrimination between the two insertions in tumor 7107 since they were so close to each other. B) Primers were designed for quantitative RT-PCR for *Il2rg* and *Med12* transcripts. The real time PCR data was normalized to thymus.
(0.59 MB TIF)

Figure S4 Tumor 7107 has one copy of *Il2rg* and two copies of *Irs2* per cell. A) Primers were designed for a segment of genomic DNA 5′ of the *Il2rg* gene and used to quantify copy number in tumor 7107. As control, genomic DNA was prepared from male and female mouse thymi. Ct values were plotted versus log (ng of gDNA). Equations and R^2^ values of the linear regression models are shown. Tumor 7107 QPCR shows similar trend to the male mouse consistent with one *Il2rg* copy per cell. Standard errors for each point represent triplicates and the experiment was performed twice. B) We applied QPCR to the other *Lmo2*-clonal tumors which were all female. Genomic DNA from tumor 3095 was unavailable. Ct values for 98031 were normalized to 1 for relative quantification. The results confirm that tumor 7107 was not polyploid for *Il2rg* gene. Standard errors are shown. Independent t-test (*p* = 5.3×10^−5^) and Mann-Whitney U-test (*p* = 0.02) both showed statistically significant difference between tumor 7107 and the other tumors. This confirms that all the tumors are diploid for chromosome X except for tumor 7107 which arose in a male mouse. C) A BAC encompassing the mouse *Irs2* gene (chromosome 8) was labeled with Spectrum Orange and fluorescent in situ hybridization (FISH) performed on paraffin-embedded tumor. The section shown is completely involved with tumor cells and shows two signals for the labeled probe per cell. We did not find hyperdiploidy for *Irs2* throughout the tumor.
(2.16 MB TIF)

Figure S5 Common gamma cytokine pathways are enriched in human and mouse T-ALL data sets. We combined human gene expression data with mouse insertion site profiles and performed Ingenuity pathway analysis. We identified a network of genes that were upregulated in at least two of the data sets. Statistical analysis of the Biocarta pathways present in this network is shown. The Ingenuity network contained 15 genes. List hits indicates how many of these were present in the specific Biocarta pathway. Population hits shows how many genes are present in the Biocarta pathway and Population total shows how many total genes are present in all of Biocarta at the time of our analysis. *P* values are calculated using Fisher's exact test.
(2.81 MB TIF)

Table S1 This table shows a list of genes that are common to mouse and human leukemias.
(0.02 MB XLS)

Text S1 Supplementary methods and results.
(0.07 MB DOC)

Dataset S1 This is a more comprehensive version of Table 1 which shows common insertion sites as well those insertions that are not CIS in the Retroviral Tagged Cancer Gene Database. If the gene has been implicated in human or mouse cancer, then the associated pathology is shown based on Sanger's Cancer Gene Census. Number of clones and frequency indicates how often an insertion appeared after shotgun cloning. It is calculated as the total identical clones divided by all informative clones found per tumor. Higher frequency indicates higher enrichment in the tumor genomic DNA.
(0.04 MB XLS)
