# Supplementary figures and images for: Murine Leukemias with Retroviral Insertions at Lmo2 Are Predictive of the Leukemias Induced in SCID-X1 Patients Following Retroviral Gene Therapy
Source: PLoS Genet. 2009 May 22;5(5):e1000491. doi: 10.1371/journal.pgen.1000491 (PMC2679194; doi:10.1371/journal.pgen.1000491)

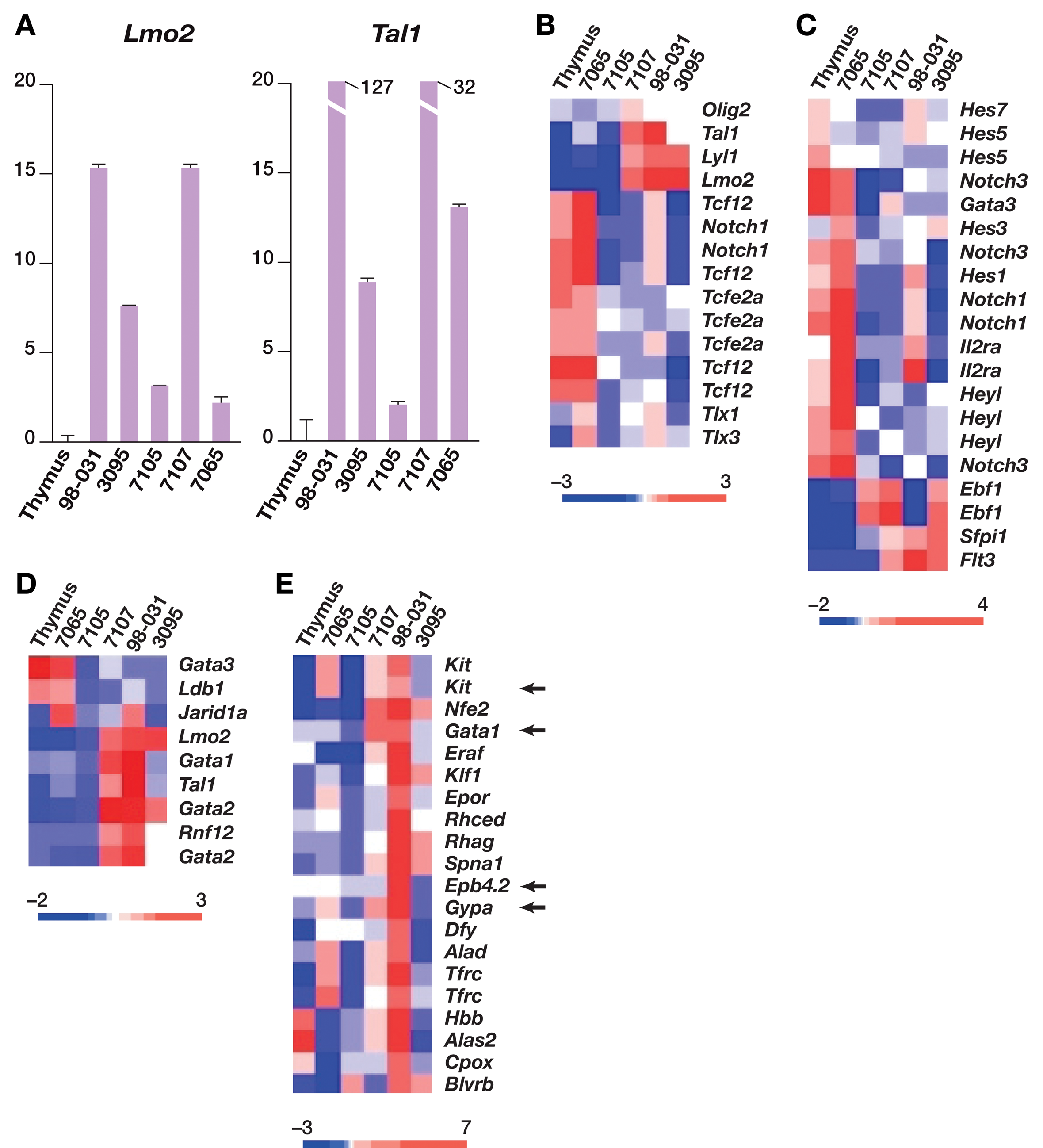

Supplement: Figure S1 — AKXD Lmo2 tumors co-express Tal1. A) quantitative RT-PCR shows Lmo2 and Tal1 are overexpressed in the tumors with respect to normal thymus. To the right are two heat maps from microarray expression data. B) This panel shows T-cell leukemia transcription factors and their expression in the Lmo2-clonal tumors. RNA from normal thymus and tumor 7065, which has a clonal, activating mutation in Notch1, are included in the comparison. Lmo2-clonal tumors have higher class II bHLH transcription factor expression and lower expression of E2A genes when compared to normal thymus and tumor 7065. C) This heat map shows documented Notch1 targets and their expression in the same tumors. All genes shown are normally up-regulated by Notch1 except for Ebf1, Sfpi1, and Flt3, which are repressed. The Lmo2-clonal tumors show low expression of Notch1 target genes and higher expression of genes that are repressed. Tumor 98031 has higher expression of Hes1 and Il2ra and lower expression of Ebf1, consistent with a heterozygous mutation in the heterodimerization domain of Notch1. The rest of the tumors had wild type Notch1 sequences. D) Experimentally confirmed Lmo2-binding partners are shown on this heat map. All of these were expressed at levels higher than normal thymus and tumor 7065. The Lmo2-clonal tumors had high expression of Gata1 and Gata2. Gata1 was very high in tumor 98031 and so we assayed the expression of erythroid genes in this tumor. E) Numerous erythroid genes were up-regulated in 98031. The genes denoted by the black arrows are activated by an Lmo2/Gata1/2/Tal1/E47/Ldb1-containing oligomeric complex that binds to promoter E box/Gata-motifs. Log2 intensity scales are shown for the heat maps. (2.29 MB TIF) [file pgen.1000491.s001.tif]

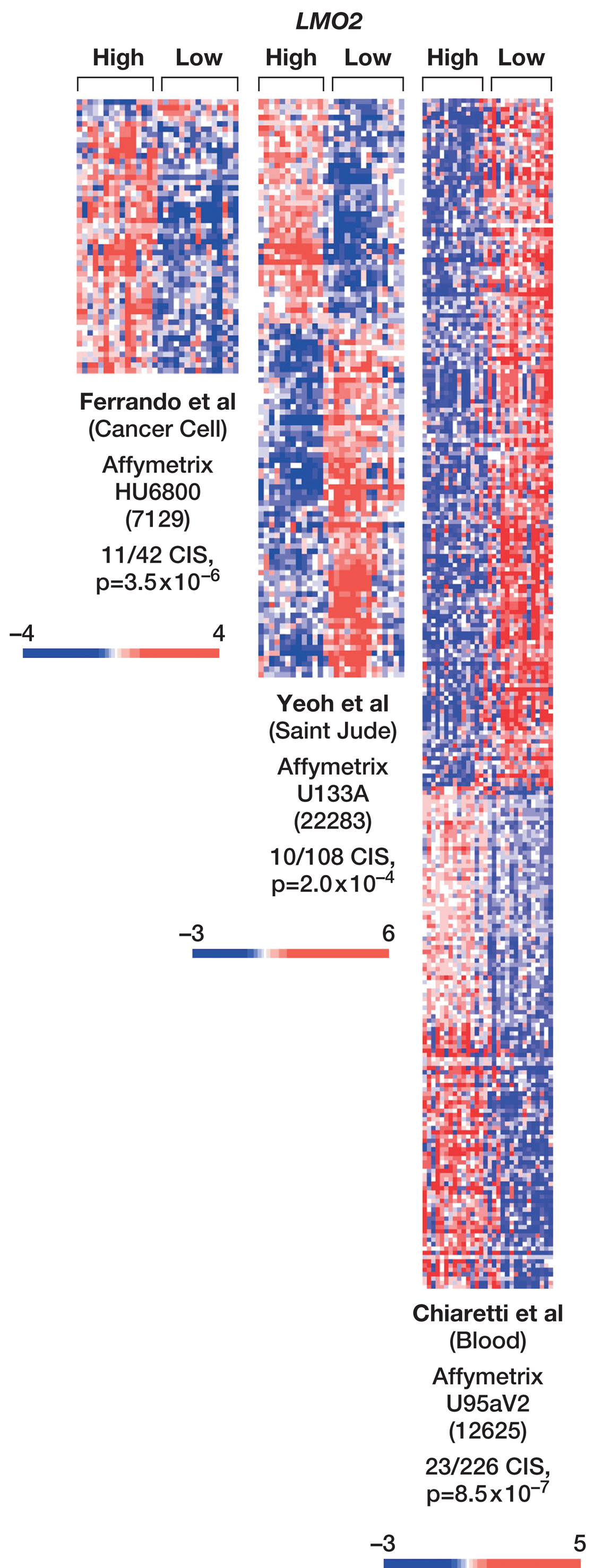

Supplement: Figure S2 — Human T-ALL microarray analysis shows representation of many CIS in the transcriptional profile of LMO2-overexpressing patients. Raw data were retrieved from three large published T-ALL studies and cases were clustered into LMO2-high expressing and LMO2-low expressing classes. We next sought to find the most statistically significant genes that clustered with the LMO2-high group. The studies' first authors are cited as well as the platforms used. In parentheses, we show how many probes were on the chip used. Below this, we show the number of CIS divided by the total number of genes that clustered with the LMO2-high expressing cases. The last number is the p value for identifying these CIS as calculated by Fisher's exact test. (2.78 MB TIF) [file pgen.1000491.s002.tif]

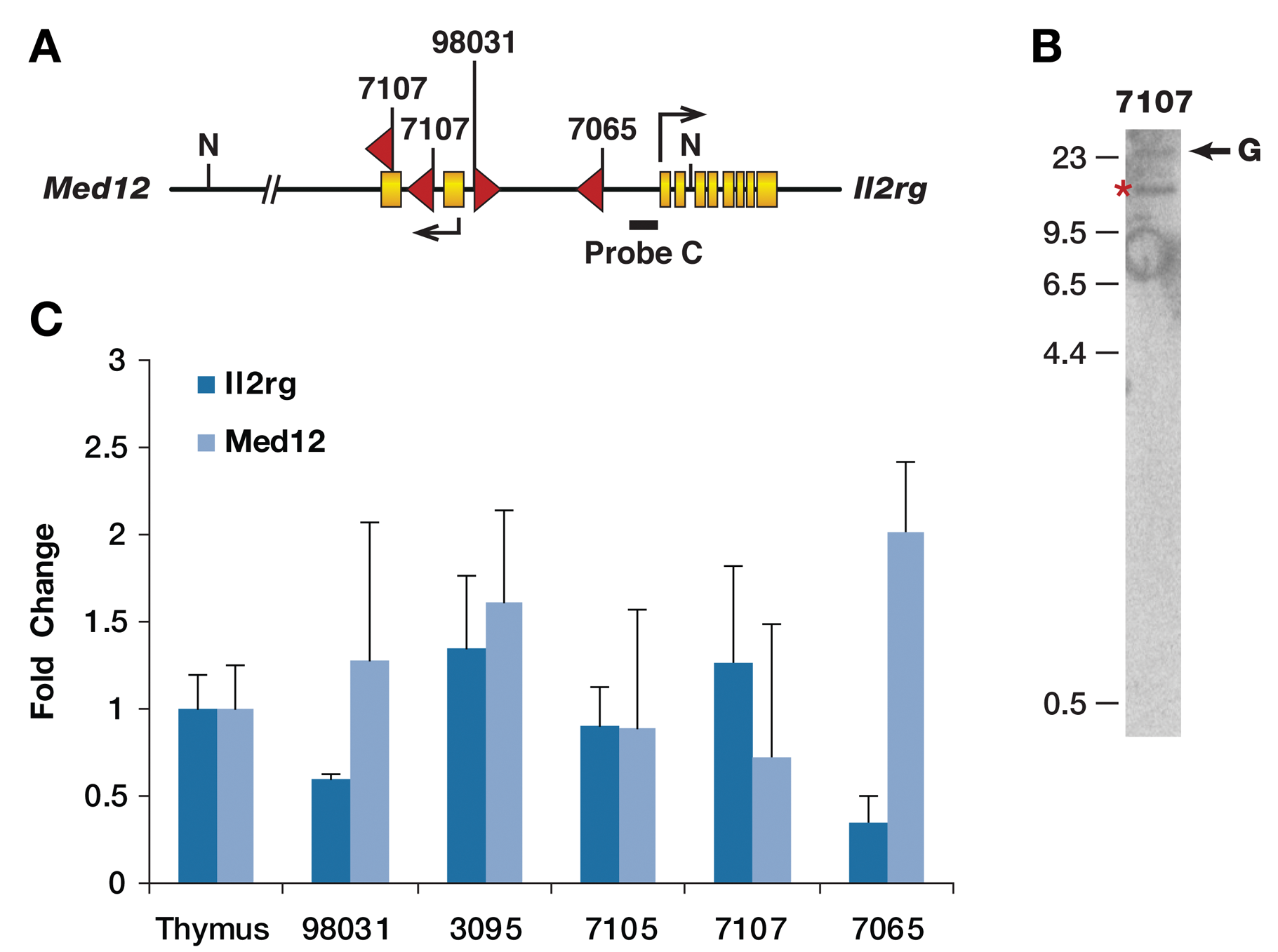

Supplement: Figure S3 — Il2rg insertions are clonal but cause no transcriptional activation. A) the structure of the genomic region with Il2rg and Med12 genes is shown. Yellow exons are coding and black arrows show transcriptional start sites and orientation. Southern analysis of tumor 7107 was done using NcoI digested tumor DNA. The germline band is identified by the arrow, G. The rearranged band is present with equal intensity and shown by the red asterisk. The size of the NcoI fragment did not allow discrimination between the two insertions in tumor 7107 since they were so close to each other. B) Primers were designed for quantitative RT-PCR for Il2rg and Med12 transcripts. The real time PCR data was normalized to thymus. (0.59 MB TIF) [file pgen.1000491.s003.tif]

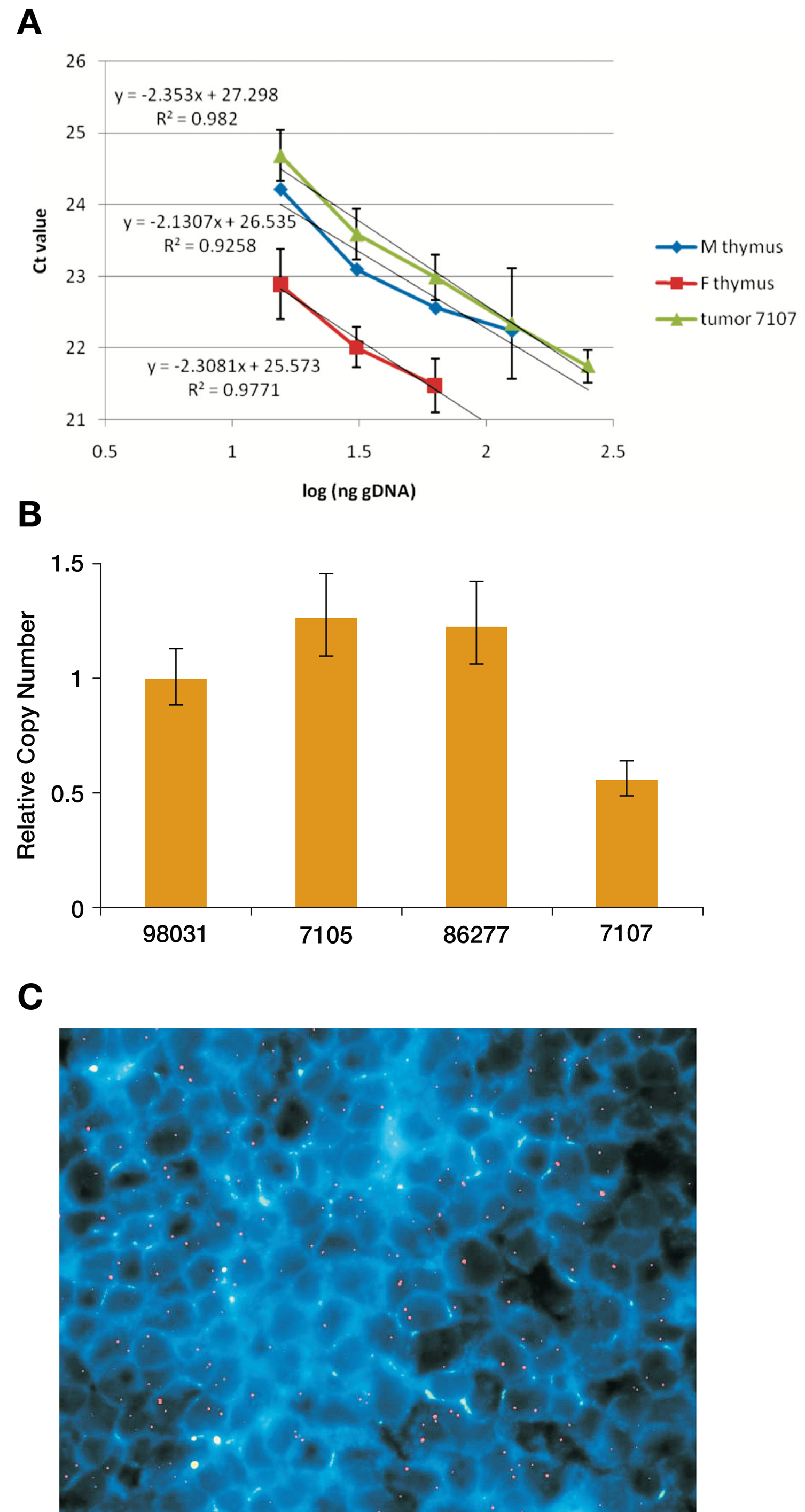

Supplement: Figure S4 — Tumor 7107 has one copy of Il2rg and two copies of Irs2 per cell. A) Primers were designed for a segment of genomic DNA 5′ of the Il2rg gene and used to quantify copy number in tumor 7107. As control, genomic DNA was prepared from male and female mouse thymi. Ct values were plotted versus log (ng of gDNA). Equations and R2 values of the linear regression models are shown. Tumor 7107 QPCR shows similar trend to the male mouse consistent with one Il2rg copy per cell. Standard errors for each point represent triplicates and the experiment was performed twice. B) We applied QPCR to the other Lmo2-clonal tumors which were all female. Genomic DNA from tumor 3095 was unavailable. Ct values for 98031 were normalized to 1 for relative quantification. The results confirm that tumor 7107 was not polyploid for Il2rg gene. Standard errors are shown. Independent t-test (p = 5.3×10^−5^) and Mann-Whitney U-test (p = 0.02) both showed statistically significant difference between tumor 7107 and the other tumors. This confirms that all the tumors are diploid for chromosome X except for tumor 7107 which arose in a male mouse. C) A BAC encompassing the mouse Irs2 gene (chromosome 8) was labeled with Spectrum Orange and fluorescent in situ hybridization (FISH) performed on paraffin-embedded tumor. The section shown is completely involved with tumor cells and shows two signals for the labeled probe per cell. We did not find hyperdiploidy for Irs2 throughout the tumor. (2.16 MB TIF) [file pgen.1000491.s004.tif]

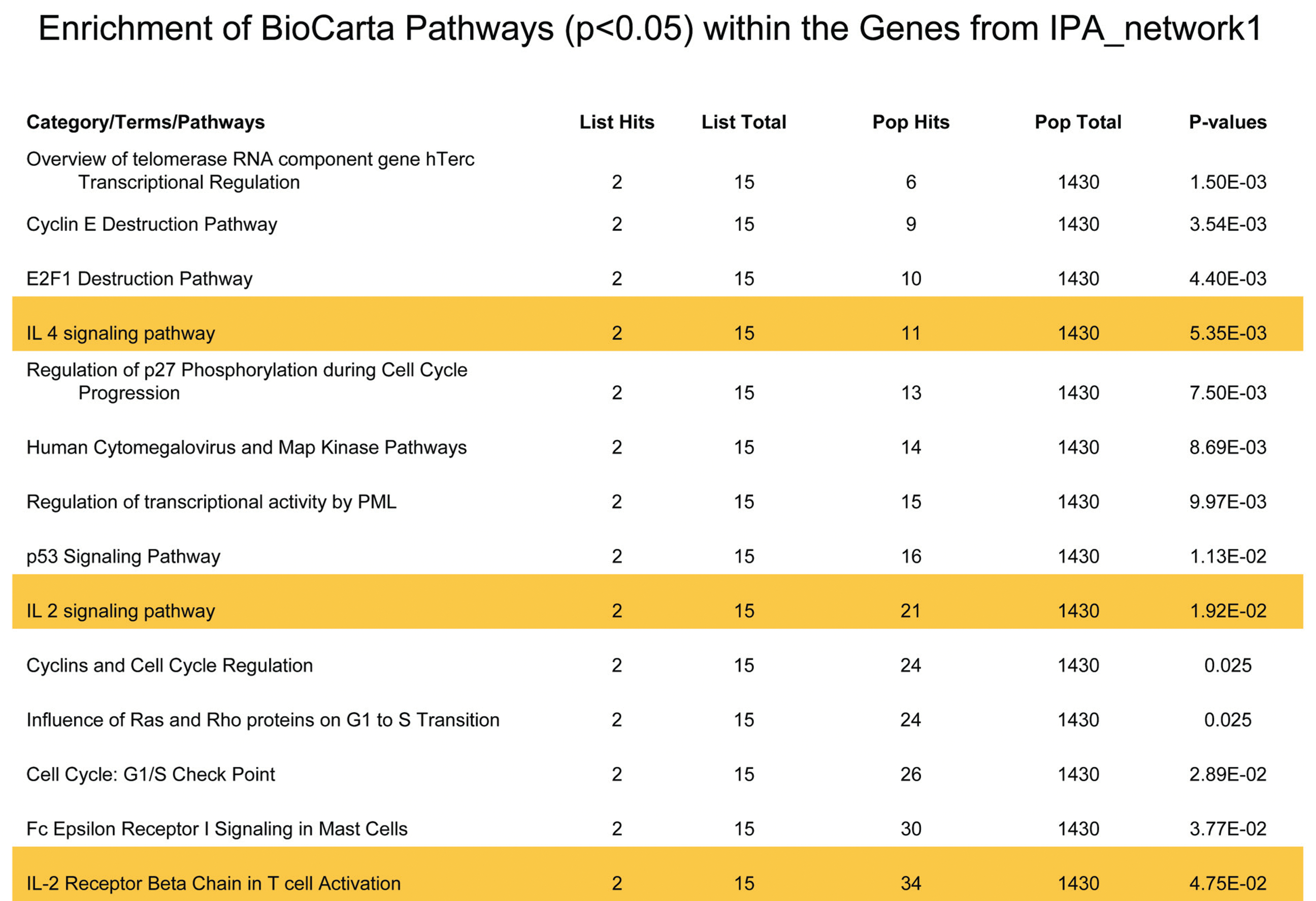

Supplement: Figure S5 — Common gamma cytokine pathways are enriched in human and mouse T-ALL data sets. We combined human gene expression data with mouse insertion site profiles and performed Ingenuity pathway analysis. We identified a network of genes that were upregulated in at least two of the data sets. Statistical analysis of the Biocarta pathways present in this network is shown. The Ingenuity network contained 15 genes. List hits indicates how many of these were present in the specific Biocarta pathway. Population hits shows how many genes are present in the Biocarta pathway and Population total shows how many total genes are present in all of Biocarta at the time of our analysis. P values are calculated using Fisher's exact test. (2.81 MB TIF) [file pgen.1000491.s005.tif]
