# Supplementary material for: Murine Leukemias with Retroviral Insertions at Lmo2 Are Predictive of the Leukemias Induced in SCID-X1 Patients Following Retroviral Gene Therapy
Source: PLoS Genet. 2009 May 22;5(5):e1000491. doi: 10.1371/journal.pgen.1000491 (PMC2679194; doi:10.1371/journal.pgen.1000491)
Supplement: Text S1 — Supplementary methods and results. (0.07 MB DOC) [file pgen.1000491.s007.doc]

**Supplementary Methods**

**Affymetrix chip hybridizations**

The Affymetrix One Cycle reaction was initiated using a sample mix of 1.5 μg high-quality total RNA, 2 μl poly-A spike in controls (included in kit), 2 μl 50uM T7 dT oligo, and H2O to 12 μl final volume, which is incubated 10 minutes at 70°C, then cooled to 4°C for 5 minutes in a thermal cycler. Next, 8μl of first strand synthesis mix (4μl 5X first strand buffer, 2μl 0.1M DTT, 1μl 10mM dNTP, and 1 μl SuperScript II) is added to the sample mix and incubated at 42°C for 1 hour, with cooling to 4°C for 5 minutes. Subsequently, 130 μl second strand synthesis mix (30 μl second strand buffer mix, 3μl 10mM dNTP mix, 1μl E. Coli DNA ligase, 4 μl E. Coli DNA polymerase I, and 1μl E. Coli Rnase H) is added to the sample mix, and incubated at 16°C for 2 hours. At this point, 2 μl of T4 DNA polymerase is introduced, and incubated at 16°C for 5 minutes, followed by the addition of 10 μl 0.5M EDTA to stop the reaction. The samples are then purified by binding to and elution from a Qiagen column. The resulting cDNA is next labeled and amplified in an IVT reaction utilizing biotinylated nucleotides. The reaction is done by adding the purified cDNA to 28μl of labeling mix (4 μl 10X IVT labeling buffer, 12 μl IVT labeling NTP mix, 4μl IVT labeling enzyme mix, and 8μl H2O) and incubating 16 hours at 37°C. The reaction products are biotinylated cRNA molecules, which are purified by binding and elution from a Qiagen column. After quantitation, 20 μg of the reaction products are fragmented using 8 μl of fragmentation buffer and H2O to a final volume of 40 μl, and incubated at 94°C for 35 minutes. Fragmentation reaction products and non-fragmented cRNA samples are compared with bioanalysis to insure that fragmentation is complete. Fragmented samples are then hybridized to the proper array, stained and scanned.

Hybridization data in CEL format was then handled as described for human T-ALL datasets.

**Pathway analysis**

The analysis of pathway and network data was performed using in-house software WPS in the context of general functional classes using Gene Ontology (GO) ([http://www.geneontology.org](http://www.geneontology.org/)) and BioCarta pathways (<http://www.biocarta.com/genes/allPathways.asp>). One-sided Fisher’s exact test *P* values were calculated to determine whether a particular pathway or GO term was statistically significantly enriched in intended gene lists. “list hits” were generated to measure how many genes from these lists hit a pathway or GO term. At the individual gene level, the defined patterned gene lists (e.g., genes differentially expressed in other *Lmo2* tumors but not in 7065 etc.) with common or different behaviors in multiple tumor samples were extracted for further analysis using color-coded based pattern extraction feature within the in-house WPS program. Then at the pathway or gene group level, the enriched GO terms or BioCarta pathways were computed by Fisher’s exact test within the WPS program using the gene lists. To compare biological themes at the pathway and GO term level enriched across multiple gene lists, they were also subjected to batch computation of using a one-sided Fisher’s exact test. The log-transformed p-values were retrieved and combined for clustering analysis to look for common or unique behaviors at pathway or GO term levels that are enriched within those lists. The GO terms or BioCarta pathways of selected clusters of interest were also used to retrieve the associated genes from the original lists.

**Statistical analysis**

**Probability of finding 2 or more tumors with at least 1 TCF family gene insertion from 5 tumors**

<Use binomial probability>

Number of TCF family genes: Mouse genes named like TCF or previously named like TCF in NCBI GenBank = 30

Total number of genes annotated and aligned mouse genome = 17000

Probability of successfully finding TCF genes in 1 trial = 30/17000 = 0.00176

Average number of insertions per tumor = 84 / 5 = 17

Finding 1 (1 success) or more TCF family genes in 1 tumor (17 trials) = 1 – cdf.binom(1-1, 17, 30/17000) = 0.02958

Finding 2 (2 success) or more tumors with TCF family genes from 5 tumors (5 trials) = 1 – cdf.binom(2 – 1, 5, 0.02958) = 0.0082435

A1: p = 0.00824

**Probability of finding Lmo2 tumor CIS genes (22 distinct CIS genes from Table 1) from CIS genes in microarray result**

Use of Chi Square test:

Number of total CIS genes in RTCGD: 500 genes (approximately)

Number of CIS genes found in Lmo2 tumors: 22 genes

Ferrando’s microarray result

Number of CIS genes: 12 genes

Number of Lmo2 tumor CIS genes: 4 genes

p-value = 0.0011113941238807834

|  | Lmo2 gene | Non-Lmo2 gene | Total |
| --- | --- | --- | --- |
| Ferrando | 4 | 8 | 12 |
| Non-Ferrando | 18 | 470 | 488 |
|  | 22 | 478 | 500 |

Yeoh’s microarray result

Number of CIS genes: 12 genes

Number of Lmo2 tumor CIS genes: 4 genes

p-value = 0.0011113941238807834

|  | Lmo2 gene | Non-Lmo2 gene | Total |
| --- | --- | --- | --- |
| Yeoh | 4 | 8 | 12 |
| Non-Yeoh | 18 | 470 | 488 |
|  | 22 | 478 | 500 |

Chiaretti’s microarray result

Number of CIS genes: 19 genes

Number of Lmo2 tumor CIS genes: 7 genes

p-value = 0.0000041728001115516134

|  | Lmo2 gene | Non-Lmo2 gene | Total |
| --- | --- | --- | --- |
| Chiaretti | 7 | 12 | 19 |
| Non-Chiaretti | 15 | 466 | 481 |
|  | 22 | 478 | 500 |

**Probability of finding common insertion sites in the LMO2-high expressing class of patients analyzed by microarray (see supplemental Figure 1).** Below, List Hits indicates how many genes are present on the microarray LMO2-high class and that are also CIS in the RTCGD. List total indicates how many total genes are in the LMO2-high class. Population Hits indicates how many CIS genes are present on the array chip. Population total indicates how many total genes are on the array. A 2x2 contingency table was constructed using these data and Fisher’s exact test used to calculate p values.

| ListHits | ListTotal | PopulationHits | PopulationTotal | **Fisher Exact P-Values** | Details |
| --- | --- | --- | --- | --- | --- |
| 23 | 226 | 337 | 9754 | 8.50E-07 | Chiaretti et al |
| 10 | 108 | 398 | 14438 | 1.99E-04 | Yeoh et al |
| 11 | 42 | 236 | 6017 | 3.46694E-06 | Ferrando et al |

**Probability of finding two tumors with insertions in Lmo2 and Il2rg**

The probability of finding two tumors with two specific genes (Lmo2 and Il2rg) is approximately 1.336E-9.

Let's assume there are five tumors with 17 independent insertion sites (total 84 insertions).

The probability of finding Lmo2 and Il2rg in one tumor is

C(17,1) * C(16,1) * C(15,15)* (1 / 20000) * (1 / 20000) * (19998 / 20000)^15 = 1.156E-5

(1 insertion from Lmo2, 1 insertion from Il2rg, and 15 insertions from the rest of 19998 genes)

Then, the probability of finding two tumors with Lmo2 and Il2rg insertions out of five tumors is

C(5,2) * (1.156E-5)^2 * (1 - 1.156E-5)^3 = 1.336E-9

(2 tumors must have Lmo2 and Il2rg, and the rest of 3 tumors do not have the insertion pattern.)  Any gene other than Il2rg will provide the same result.

**SupplementaRY RESULTS AND FIGURES**

**The *Lmo2* interacting gene *Tal1* is coexpressed with *Lmo2* in AKXD *Lmo2* tumors**

Lmo2 contains 2 LIM zinc finger-like protein interaction domains and acts as a bridge protein between Tal1 (Scl)/Tcfe2a heterodimers and Gata1/2/3 proteins [1]. *TAL1* and *LMO2* are co-mutated in a subset of human leukemias, suggesting they promote leukemia cooperatively [2]. Quantitative RT-PCR showed that the four AKXD *Lmo2* tumors for which flash frozen tissue was available overexpressed *Tal1* and *Lmo2* (R^2^=0.92, p=0.041) (Figure 5A). Analysis of an additional 18 retrovirally induced T-cell tumors that lacked *Lmo2* insertions also identified a statistically significant concordant expression between *Lmo2* and *Tal1* (N=22, R^2^=0.66, p=3.98 x 10^-6^). Thus, *Tal1* is co-expressed with *Lmo2* in mouse and human tumors. In control experiments, we analyzed RNA from tumor 7065, which had a clonal insertion in *Notch1* but not *Lmo2*. This tumor had elevated levels of *Lmo2*, but not to the same degree as tumors with *Lmo2* insertions (Figure 5A).

**Other Lmo2 binding partners are also expressed in AKXD *Lmo2* tumors**

We used an Affymetrix chip to identify additional genes whose expression might also be deregulated in AKXD *Lmo2* tumors. These tumors showed lower expression of *Tcfe2a* and *Tcf12*, and higher expression of *Tal1* and *Lyl1*, compared to controls (Figure 5B), consistent with the quantitative RT-PCR experiments. With the exception of tumor 98-031, most *Notch1* targets were poorly expressed in these tumors (Figure 5C). Tumor 7065 showed the highest expression of *Notch1* target genes consistent with it having an activating viral insertion in *Notch1* [3]. Sequence analysis of *Notch1* exons in the *Lmo2*-clonal tumors showed a putative activating mutation in the heterodimerization domain (Leu1569Pro) of only one tumor, 98031, consistent with its high expression of Notch1 target genes, *Il2ra* and *Hes1* (data not shown)[3].

All experimentally confirmed *Lmo2* binding partners were expressed in the AKXD *Lmo2* tumors (Figure 5D). Interestingly, *Gata3*, a T-cell-specific transcription factor and transcriptional target of *Notch1*, was not expressed in AKXD *Lmo2* tumors, whereas *Gata1* and *Gata2* were highly expressed. Lmo1/2 proteins only bind DNA when complexed with Gata transcription factors (1-3) and Tcfe2a/Tal1 heterodimers. This complex is stabilized by LIM-domain binding-1 (*Ldb1*) protein and assembles over an E-box-Gata motif that is specifically spaced in the promoters of target genes [4]. Tumor 98-031 was unique in its very high level of expression of *Gata1* (7.3-fold higher than thymus), a gene known to be required for erythroid and megakaryocyte differentiation, so we looked at erythroid transcripts in this tumor and found a cluster that was highly upregulated, which included confirmed targets of the Lmo2/Tal1/Ldb1/Gata1/Tcfe2a oligomeric complex (black arrows, Figure 5E). *Gata1* and *Gata2* were also highly expressed in tumor 7107 although not to the degree seen in tumor 98-031. Tumor 7107 also showed upregulated expression of *Kit* and *Gypa* consistent with an active oligomeric complex containing the Lmo2 protein.

**A role for cytokine signaling in AKXD *Lmo2* tumors**

The upregulation in AKXD *Lmo2* tumors of the Lyn and Syk Src family kinases, which transduce signals from cytokine receptor complexes like IL-2R and IL-7R, and the frequent insertions in *Il2rg* in AKXD *Lmo2* tumors, also suggest a role for cytokine signaling in these tumors. Supporting this, Ingenuity pathway analysis performed on genes that are shared in at least two data sets (mouse and human combined), identified a network that is highly enriched in Biocarta pathways related to cytokine signal transduction in tumors with upregulated *Lmo2* expression (see Supplemental Figure 4): IL-4 signaling (p=5.35 x 10 ^-3^), IL-2 signaling (p=1.92 x 10^-2^) and IL-2 receptor beta chain signaling in T cell activation (p=4.75 x 10^-2^).

**REFERENCES**

1. Nam CH, Rabbitts TH (2006) The role of LMO2 in development and in T cell leukemia after chromosomal translocation or retroviral insertion. Mol Ther 13: 15-25.

2. Larson RC, Osada H, Larson TA, Lavenir I, Rabbitts TH (1995) The oncogenic LIM protein Rbtn2 causes thymic developmental aberrations that precede malignancy in transgenic mice. Oncogene 11: 853-862.

3. Weng AP, Ferrando AA, Lee W, Morris JPt, Silverman LB, et al. (2004) Activating mutations of NOTCH1 in human T cell acute lymphoblastic leukemia. Science 306: 269-271.

4. Wadman IA, Osada H, Grutz GG, Agulnick AD, Westphal H, et al. (1997) The LIM-only protein Lmo2 is a bridging molecule assembling an erythroid, DNA-binding complex which includes the TAL1, E47, GATA-1 and Ldb1/NLI proteins. Embo J 16: 3145-3157.
